# Human Microphysiological Systems and Organoids as *in Vitro* Models for Toxicological Studies

**DOI:** 10.3389/fpubh.2018.00185

**Published:** 2018-07-10

**Authors:** George A. Truskey

**Affiliations:** Department of Biomedical Engineering, Duke University, Durham, NC, United States

**Keywords:** microphysiological systems, organoids, toxicity, environmental pollutants, cell culture, stem cells

## Abstract

Organoids and microphysiological systems represent two current approaches to reproduce organ function *in vitro*. These systems can potentially provide unbiased assays of function which are needed to understand the mechanism of action of environmental toxins. Culture models that replicate organ function and interactions among cell types and tissues move beyond existing screens that target individual pathways and provide a means to assay context-dependent function. The current state of organoid cultures and microphysiological systems is reviewed and applications discussed. While few studies have examined environmental pollutants, studies with drugs demonstrate the power of these systems to assess toxicity as well as mechanism of action. Strengths and limitations of organoids and microphysiological systems are reviewed and challenges are identified to produce suitable high capacity functional assays.

## Introduction

Animal toxicological studies provide important information about the effects of a range of drugs, toxins and pollutants. Animal models of disease often replicate some key symptoms but do not reproduce the pathology and thus do not accurately predict the response in humans. While scaling relationships between species and physiologically-based pharmacokinetic models provide important information to predict safety and efficacy in humans (1), the expression levels and activities of key liver enzymes (2) and renal transporters (3) differ between humans and rodents, which are used in many drug distribution studies. In many cases, a transgenic mouse may be the only pre-clinical model. However, a transgenic mouse model often does not match the severity or phenotype or share the same genetic basis of the related human disease. Further, rodent physiology is drastically different from that in humans and other large animals. For example, humans and mice exhibit different transcriptional responses to inflammatory diseases and variations among mouse strains are significant and unrelated (4). Animal models are of limited use when studying the response to environmental toxins in individuals who may be more susceptible than the general population, such as in the developing fetus, children, the elderly and those with diseases.

Environmental pollutants act by modulating or interfering with biochemical pathways or modifying DNA. These changes then activate the immune system, induce cancer, cause neurotoxicity, or produce birth defects or developmental disorders. The number of environmental pollutants is quite extensive and growing, while only a few have been adequately studied. To cope with the large number of unevaluated chemicals in the environment, US and European agencies have initiated a concerted effort by to develop better tools to screen compounds for potentially harmful biological effects. While carefully planned high throughput screening can produce important insights about the model of action of pollutants (5), the lack of success of targeted screens to identify new drug candidates suggest that phenotypic approaches are needed to develop a complete understanding of the mechanism by which pollutants induce pathology (6).

A number of *in vitro* two-dimensional cell culture systems have been used to study toxicological responses and three-dimensional systems are used for specialized applications including spheroids, hanging drop (7), and Transwell systems (8). In general, most of these simpler systems have limited utility for drug discovery and toxicology due to several limitations (9). The culture media is often not optimized to promote differentiation. Cells do not differentiate well due to the mechanical rigidity of tissue culture polystyrene and the absence of mechanical forces (9). In tissues, multiple cell types are present and coculture conditions are needed to promote optimal function of cells (7, 9).

Several features are critical to developing useful cell culture models to assess disease mechanisms of drugs as well as environmental pollutants (6). While cell lines have the advantage of reproducibility and ease of access, cell lines are transformed, and cell signaling pathways and function differ from those in the native cells. Thus, primary cells in early passage should be used and their extent of differentiation should be validated. Further, functional assays that measure one or more important physiological variables should be used in addition to gene or protein expression (6). The signaling pathways and responses activated in response to drugs, toxins, or pollutants are often very dependent on the local environment, strongly affecting the manner in which a drug or environmental chemical affects the response (10).

To address these limitations, recent advances in cell culture technology, stem cell biology, biomaterials, microfluidics and biosensors have been applied to the development of human organoids (11) and human microphysiological systems (or organs-on-a-chip) (12). While still in the early stages of development these technologies offer the potential to provide better *in vitro* models to perform toxicological studies by more closely modeling the *in vivo* environment. Advances in methods to produce differentiated cells and tissues from human pluripotent stem (hPS) cells or human induced pluripotent stem (iPS) cells, as well as transdifferentiation from one cell type to another offer new opportunities to develop *in vitro* disease models to study mechanisms and develop new therapies.

In this paper, we review and compare human organoid and microphysiological systems, summarize their application in toxicity studies and discuss advances needed to be used in a predictive manner for toxicological studies of environmental pollutants.

## Organoid systems

**Figure 1:**
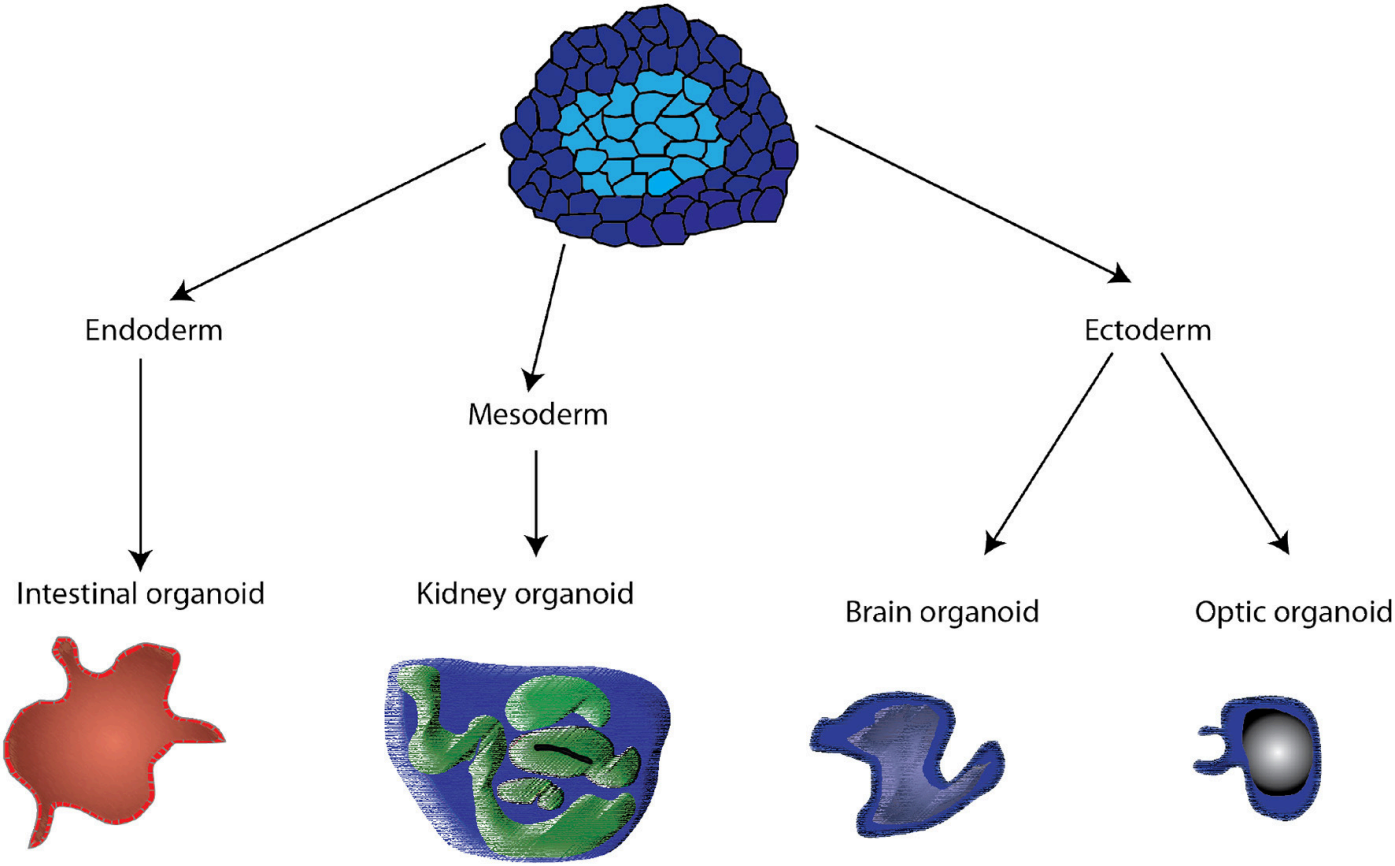
Schematic of the different pathways by which organoids are derived from pluripotent stem cells. Control of media and the addition of small molecules activing specific transcription factors drive the stem cells to endoderm, mesoderm, and ectoderm. Subsequent treatments are then used to produce specific organoids.

Organoids are *in vitro* tissue models that consist of “a collection of organ-specific cell types that develops from stem cells or organ progenitors and self-organizes through cell sorting and spatially restricted lineage commitment in a manner similar to *in vivo*” (13). Organoids have been produced using adult, embryonic and induced pluripotent stem cells. The stem or progenitor cells undergo division, often in a biological hydrogel matrix, such as Matrigel, and through a process of genetically-encoded self-organization (14), cells with different adhesion receptors, associate with each other and produce a three-dimensional structure often representing the functional unit of a tissue (Figure 1). Organoids can be produced by directed or undirected differentiation (15). In directed differentiation, hPS induced to a specific lineage (endoderm, mesoderm, or ectoderm) are then driven to a specific tissue phenotype using small molecular regulators of transcription factors in tissue-specific media, patterning, three-dimensional culture in hydrogels or suspension. For brain organoids, directed differentiation produced specific brain regions (15). In undirected differentiation, the hPS cells are incubated with culture media containing tissue-specific growth factors and hormones, but without small molecules that activate specific lineage pathways. The latter approach works best with tissues that are relatively uniform containing one or two different cell types; otherwise more complex tissue, like brain (15), exhibit reduced specialization and reduced organization and require more precise differentiation. When properly differentiated, the organoid reproduces the structure of the tissue or a functional unit of the tissue, with tissue-specific molecules being produced, and some limited organ function can be produced. Often organoids can be maintained in culture for months when cultured with activators of specific pathways (16).

### Examples of organoid systems

Murine organoids have been obtained for a large number of tissues (13). Human organoid systems have been developed for the fallopian tubes (16), small intestine (17), retina (18), cornea (19), brain (15), liver (20, 21), pancreas (22), prostate (23), salivary glands (24), lung (8), kidney (25), and endometrium (26). Using adult stem cells, organ progenitor cells or iPS cells, organoids can be developed that represent the diversity of a population to examine various mutations that increase susceptibility to disease. Human iPS cells can be differentiated into specific tissues to study disease development (13), cancer (27), infections of brain and gut-microbiome organoids (28), and generate models of cystic fibrosis (29). CRISPR technology can be used to test models of cancer development or test the effectiveness of proposed corrections of genetic diseases (30).

### Current limitations of organoid systems

Several challenges exist in using organoid cultures to assess environmental toxins. The organoids tend to form clumps or spherical structures with internal cavities, often altering the anatomic structure *in vivo*. This limits the ability to make functional measurements when the native tissue structure involves perfusion and/or secretion, a challenging issue for epithelial transport in organoids derived from the intestine and kidney. One approach to address this limitation is to culture organoids in a two-dimensional format on Transwell membranes as has been done with gut enteroids(17) or lung organoids (8), or to differentiate the organoids in microfluidic channels, replicating the normal tissue organization (31). The addition of endothelium and mesenchymal stem cells within a developing organoid (32) or to small numbers of established organoids (33) has improved function *in vitro* and led to rapid vascularization and function *in vivo* (32, 33).

Many organoids consist of one or two cell types and don't reflect the complete organization of the tissue. Due to the limited number of cell types arising in the organoids, resident immune cells are often missing (11), limiting studies of inflammation. Although organoids follow a developmental pathway, their size and organization tend to be variable, affecting functional measures.

Several systematic approaches have identified fabrication and culture conditions to result in more uniform structures and reduce the overall heterogeneity of the organoid population (11), while sorting gut epithelial spheroids by size, produced more uniform intestinal organoids (34). While the organoids display some mature tissue functions, they represent an early developmental stage. For instance, gut enteroids do not differentiate into the intestinal segments, do not display peristalsis and have a limited ability to form villus structures (17). As such, organoids may be a suitable model for studies of environmental toxins during early development.

## Microphysiological systems

Advances in tissue engineering, microfabrication, microfluidics, and biosensors have enabled the *in vitro* fabrication of a tissue or organ by organizing primary human cells or differentiated cells from hPS cells in a spatial arrangement similar to that observed *in vivo*. The microphysiological system is a smaller scale recapitulation of the functional unit of an organ or tissue. The ideal human microphysiological system has the following characteristics (35): (1) multicellular architecture similar to the native tissue; (2) functional representation of normal human biology; (3) reproducible and viable operation under physiological conditions for multiple weeks; (4) key features of normal and disease phenotypes; (5) range of functional behavior that represents the population diversity; (6) capable of integrating with other organ systems; and (7) amenable to high content screening for repeated dose efficacy testing, and for toxicology [absorption, distribution, metabolism, excretion and toxicity (ADMET)] and safety screening. Some, but not all of these features have been met. Human microphysiological systems have been developed for the heart (36, 37), liver (38, 39), kidney glomerulus (40), kidney proximal tubule (41), female reproductive tract (42), placental barrier (43, 44), heart (45–47), skeletal muscle (48), blood vessel (49), white adipose tissue (50), microvasculature (51), and blood-brain barrier (36, 37).

### Design considerations in microphysiological systems

**Figure 2:**
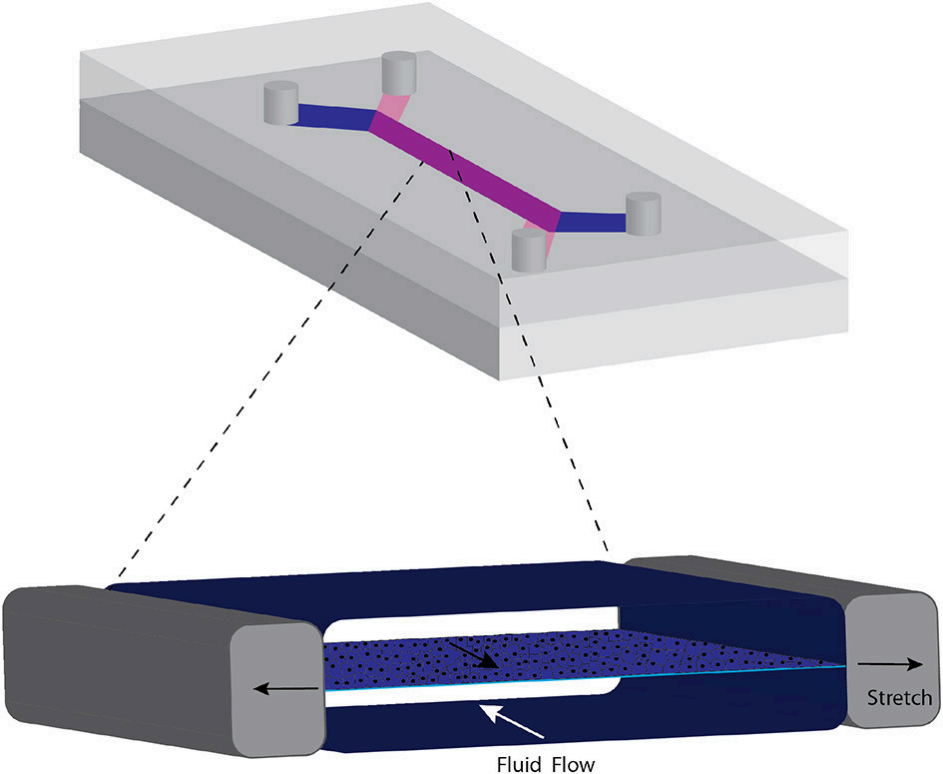
Common format of microphysiological chip for transport across barrier membranes such as those occurring in the lung and intestines. A porous elastic membrane separates the two flow streams and cells can be grown on either side. Negative pressure created in the side chambers stretches the membrane, mimicking mechanical deformation of the membrane. The cycle of stretching is varied to represent breathing or peristaltic motion.

The functional unit of organs such as lung, liver, kidney blood vessel, placental barrier, and gastrointestinal tract consist of transport across an epithelial layer separating a cavity from capillaries. The basic design that mimics this barrier region consists of a thin, porous deformable membrane separating two channels (37, 52–54). Epithelial cells form a monolayer on one side and an endothelial monolayer on the other side, representing the plasma compartment (Figure 2). For the blood-brain barrier, a confluent layer of endothelial cells are on the luminal side in contact with blood or blood mimicking media, with astrocytes on the side in contact with brain tissue (37). The fluid composition and flow rates of each channel can be controlled separately. The walls of the channels are often made from polydimethylsiloxane (PDMS), a biocompatible, transparent, and deformable polymer used in the fabrication of microfluidic devices. Some groups have used a vacuum system to expand the channels and stretch the membrane to mimic stretch that occurs in the lung during breathing (55) or peristalsis in the intestine (54). Often, other cell types are interspersed with the epithelial layer reflecting the composition of the tissue. For example, the lung airway consists of a mixture of epithelial cells that are functionally differentiated in terms of presence of cilia, secretion of mucus (goblet cells) and glycosaminoglycans (club cells) and neuroendocrine cells, overlaying stem cells (basal cells) (56). Differentiation of the epithelial layer is promoted by using the appropriate fluid, such as air for airway epithelia (56).

The immune function in the liver has been addressed by incorporating cryopreserved human Kupfer cells (38) or the U937 human monocytic cell line (39). Exposure of the liver microphysiological system after 7 days of operation with the bacterial coat lipopolysaccharide (LPS) resulted in substantial production of TNFα (38, 39), as well as the cytokines G-CSF, RANTES, IL-8, and IL-6, which are known to be associated with LPS-induced inflammation (38). Such inflammatory responses increased the cytotoxicity of drugs (39) which may help to explain acute drug-induced liver toxicity (57) and may increase the toxicity of environmental pollutants.

Mechanical forces play a critical role in promoting differentiation of cells in microphysiological systems. In a model of the lung alveolus using the design in Figure 2, mechanical stretching of the membrane to which the endothelial cells and epithelial cells enhanced the inflammatory response and generation of reactive oxygen species (55). Following treatment of the alveolar chip model with IL-2 to model pulmonary inflammation, mechanical strain enhanced the increase in permeability due to formation of gaps between the cells and fluid accumulation on the epithelial side, leading reduced oxygen saturation (58). In a gut model that stretches the epithelial layer using an arrangement similar to that shown in Figure 2 with just gut epithelial cells, applying 10% cell strain at 0.15 Hz caused the Caco-2 human epithelial cell line to form villi with columnar epithelium and a brush border and prevented a noncommensal bacteria from disrupting the epithelial barrier (54). Microarray analysis showed that these differentiated cells cultured with gut microbes were closer to human ileum than were cells cultured on a Transwell insert (54). Likewise, exposure of mouse ovarian tissue to flow in microfluidic chambers enhanced follicle maturation of ovarian follicles and secretion of follicular hormones (42).

Functional measures of microphysiological systems demonstrate that they can reproduce the behaviors of native tissue. Transendothelial electrical resistance values approach those occurring *in vivo* for the blood-brain barrier (36, 37) or gut (54). Many drugs are metabolized by cytochrome P450 (59) and measurement of its activity provides a suitable functional assessment of liver microphysiological systems. By controlling oxygen tension, cytochrome P450 activity is greater in the low oxygen concentration region of the acinus, as occurs *in vivo* (60).

Contractile force represents a functional measure of skeletal (48) and cardiac muscle (47) systems and oxygen consumption has been characterized in a skeletal muscle microphysiological system (61). Skeletal, smooth and cardiac muscle attached to deformable thin films have provided data on contractile stresses (62, 63) and contraction and dilation in the presence of agonists provides a measure of vasoactivity (49). Conduction velocity and action potential duration provide important information about the electrophysiological properties of cardiac muscle microphysiological systems that can be compared with *in vivo* behavior (47).

Organoids have been integrated into microfluidic devices to take advantage of the tissue organization that occurs with organoids and the polarity induced by membrane designs. Intestinal organoids from human duodenum biopsies were expanded and then integrated into a microfluidic channel as in Figure 2. Then the organoids were differentiated under mechanical stimulation to form villous structures that contained all of the intestinal cell types, exhibited barrier function and exhibited gene expression profiles closer to human intestine than microphysiological systems using Caco-2 cell lines (31). The advantage of this approach is that the cells in the organoid are positioned in the proper arrangement and the microfluidic system provides flow and mechanical stimulation that can promote further differentiation of the cells.

**Figure 3:**
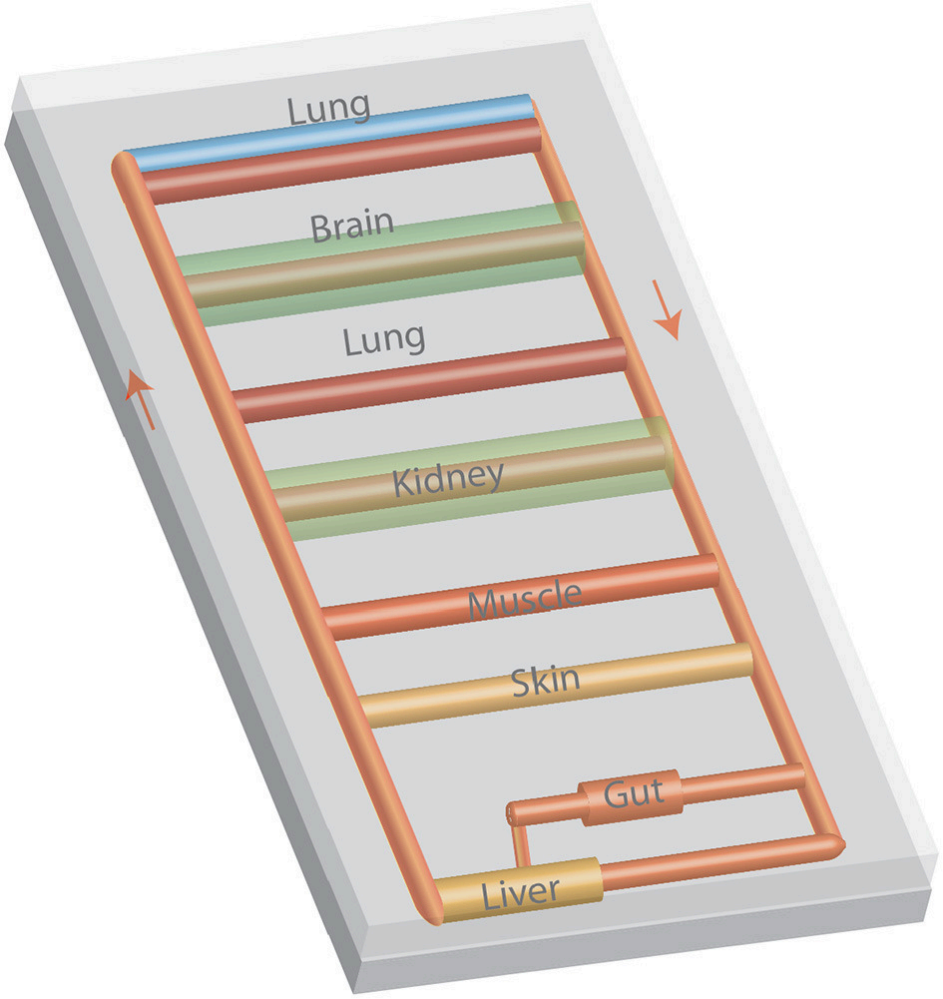
A multiple organ-on-a-chip with eight organ models. The tubing represents blood vessels and arrows represent the flow of culture media. Media can be moved with a variety of pneumatic or electromagnetic pumps or with a modification of design with a rocker to induce flow by gravity.

Multiple microphysiological systems have been interlinked to replicate interactions among various organs (Figure 3). System integration has ranged from two to ten organs. While a number of scaling approaches have been considered (64–66), one organ serves as a reference and tissue mass (or volume assuming a tissue density slightly greater than the density of water) is scaled based on body proportions. Flow rates to organs are based on the distribution of flow throughout the body and the tissue residence time (67, 68). Many systems used a closed format that resembles the body's blood circulation with a central mixing region that enables gas exchange (50, 68). Alternatively, an open format in which the media in each organ is exposed to sterile air ensures efficient gas exchange for each organ (67). By coupling gut and liver models, first pass metabolism was demonstrated (69, 70), and the contribution of each organ and organ-organ interactions assessed (69). Adding the kidney enables assessment of absorption, metabolism and excretion. The resulting clearance values for a number of drugs are similar to those reported *in vivo* (65). Linking other organs to the gut-liver-kidney modules enabled assessment of vitamin D3 transport and metabolism, blood-brain transport, skeletal muscle toxicity, and biodistribution (65). Ten (67) and thirteen (71) organ systems have been developed indicating that many of the features of the entire human body can be replicated in microfluidic systems.

### Challenges in developing microphysiological systems

Several factors influence the design and development of individual organ systems and integrated multi-organ microphysiological systems. While pumps are often used to link multiple organs in microfluidic devices, tubing volumes must be limited in order to maintain the correct relationship among the fluid and tissue volumes. Pneumatic (67, 69) or electromagnetic (42) pumps built into the multiorgan units can reduce the need for additional tubing. Alternatively, rocking platforms that utilize gravity to generate flow (72) and robotic fluid handlers (73).

Hydrophobic molecules are more likely to adsorb to PDMS, with a dramatic change in adsorption for an octanol/water partition coefficient greater than 300 (74). Drug loss of molecules with high partition coefficients can be minimized if the contact time is minimized (75), although this may not be practical given other constraints on flow and system dimensions. Drug and pollutant binding to PDMS can be reduced with a variety of coatings (74, 76), although binding should be assessed for specific molecules under study. Other materials such as polysulfones (67), polycarbonates and thermoplastics (77) exhibit low drug binding and can be micromachined to form chambers for microphysiological systems.

To increase throughput, multiple replicate, functional tubular (78) or microvascular (51) systems have been developed to increase throughput for screening, but most systems are below a 96 well format.

## The response of microphysiological systems and organoids to xenobiotics and pollutants

Most studies with microphysiological systems have focused on examining drugs for toxicity and, in some cases, efficacy. Human primary of hPS cells are necessary to assess toxicity, given the shortcomings of animal models. Various drugs that affect cardiac contractility strength and duration exerted similar effects on cardiac microphysiological systems formed using iPS-derived cardiomyocytes as determined by beat frequency (46, 79) or contractile stress (80); EC50 values were within a factor of two of values obtained with cardiac muscle. A number of drugs with known liver toxicity have been examined in several liver microphysiological systems. In a four-organ system, the gut and liver metabolized the toxic terfenadine to its nontoxic and vasoactive form fexofenadine which was unable to cross the blood-brain barrier (70). A four organ system, consisting of human cardiac, liver, skeletal muscle and neuronal cultures using a common defined medium for 2 weeks, demonstrated that differential responsiveness to a 48 h exposure to 5 μM doxorubicin (68). Consistent with reports for humans, the viability of cardiac and liver cells was reduced whereas for skeletal muscle contraction was affected, but not viability (68). Skeletal muscle systems exhibited toxicity to cerivastatin below 1 nM, which was removed the market, whereas doses above pharmacological levels were needed before toxicity to lovastatin was observed (81).

Only a few studies have examined the effect of environmental pollutants. An airway model was used to model smoking by linking the microphysiological system to a device that mimicked inhaling and exhaling cigarette smoke (52). Cigarette smoking reduces mucus clearance from the lung, due to altered function of cilia. In this model, smoking did not affect the average cilia beat frequency, but altered the distribution of beat frequencies which may alter mucus flow. Further, epithelial from patients with chronic obstructive pulmonary disease exhibited large increases in IL-8 secretion after exposure to cigarette smoke in this system, whereas epithelium from healthy nonsmoking donors did not exhibit IL-8 secretion. Interestingly, the device detected differences in the response of the airway epithelium between conventional cigarettes and e-cigarettes (52).

Brain organoids were cultured on microfluidic channels to enable perfusion and then the response to nicotine was tested (82). Nicotine exposure at levels occurring with heavy smokers inhibited differentiation and organization of the organoids which the authors concluded was similar to impaired neurogenesis observed in the fetal brain of mothers who were heavy smokers (82). Recently, dose-response toxicity studies with organoids formed with hPSCs differentiated to cardiac myocytes or primary liver cells indicated that toxicity to lead, mercury, glyphosate, and mercury, but at higher doses than observed with cultured cells or animal toxicity studies (83). This difference in toxicity may reflect a combination of the assays used, the extent of differentiation of the organoids, and the short duration of exposure (24-48 h).

Aristolochic acids (AA) are widely used in herbal teas but are implicated in kidney disease and kidney and liver cancers. Combining liver and kidney proximal tubule microphysiological systems showed that the liver modified AA-1 to produce a metabolite that was more toxic to kidney epithelial cells (84). Enzymes that modify AA-1 were identified and organic ion transporters were shown to be involved in uptake by kidney proximal tubules (84). The study showed the usefulness of microphysiological systems to identify the manner in which a naturally occurring toxin is metabolized and transported into target cells.

## Perspective

Organoids and microphysiological systems offer the potential to identify and characterize the biological impact of environmental pollutants. These systems move beyond existing screens that target individual pathways and provide a means to assay context-dependent function.

Critical issues include developing a common media, maintaining the relevant size relationship among tissues, producing the differentiated state of cells, replicating the *in vivo* organization of tissues and organs, developing more robust organoid culture systems, increasing throughput, and testing population variability. Isolation of primary cells can be challenging and limiting when doing scale-up for higher throughput screening (85). While hPS cells provide a ready source of cells, the differentiation protocols need further improvement to ensure a well-differentiated state that can maintain function following subculture for a wide range of tissues. Linking organoids and micromachined devices represents an opportunity to produce an organ system that more closely resembles the *in vivo* organ. To effectively screen large numbers of environmental pollutants, protocols or systems are needed to enable higher throughput testing. This involves identifying critical features that measure key functions of the tissue and the development of low cost sensor systems, establishing uniform methods to access and perfuse different organ systems, and integration with existing lab automation systems (85).

While multi-organ systems can replicate the entire range of absorption, disposition, metabolism and excretion (77), current microphysiological systems are complex, limiting scaling for high throughput assays. There is not likely to be a universal system useful for all applications. Rather, for the particular topic of study, the key function output need to be identified and the system designed accordingly.

Promising solutions developed to create individual and multi-organ systems with human cells suggest that the field is likely to develop rapidly over the next decade, making available new approaches to assess environmental pollutants.

## Author contributions

GT conceived of this topic and wrote the review.

### Conflict of interest statement

The author declares that the research was conducted in the absence of any commercial or financial relationships that could be construed as a potential conflict of interest.
